# A phase I study of docetaxel plus synthetic lycopene in metastatic prostate cancer patients

**DOI:** 10.1002/ctm2.1627

**Published:** 2024-03-21

**Authors:** Michael B. Lilly, Chunli Wu, Yu Ke, Wen‐Pin Chen, Adam C. Soloff, Kent Armeson, Noriko N. Yokoyama, Xiaotian Li, Liankun Song, Ying Yuan, Christine E. McLaren, Xiaolin Zi

**Affiliations:** ^1^ Hollings Cancer Center Medical University of South Carolina Charleston South Carolina USA; ^2^ Department of Urology University of California Irvine California USA; ^3^ Chao Family Comprehensive Cancer Center University of California Irvine California USA; ^4^ Department of Cardiothoracic Surgery University of Pittsburgh Pittsburgh Pennsylvania USA; ^5^ UPMC Hillman Cancer Center Pittsburgh Pennsylvania USA; ^6^ Department of Biostatistics University of Texas, MD Anderson Cancer Center Houston Texas USA; ^7^ Department of Epidemiology University of California Irvine California USA; ^8^ Veterans Affairs Long Beach Healthcare System Long Beach California USA

**Keywords:** docetaxel, lycopene, phase I trial, prostate cancer

## Abstract

**Purpose:**

Our preclinical studies showed that lycopene enhanced the anti‐prostate cancer efficacy of docetaxel in animal models. A phase I trial (NCT0149519) was conducted to identify an optimum dose of synthetic lycopene in combination with docetaxel (and androgen blockade [androgen deprivation therapy, ADT]), and to evaluate its effect on the safety and pharmacokinetics of docetaxel in men with metastatic prostate cancer.

**Methods:**

Subjects were treated with 21‐day cycles of 75 mg/m^2^ docetaxel (and ADT), plus lycopene at 30, 90 or 150 mg/day. A Bayesian model averaging continual reassessment method was used to guide dose escalation. Pharmacokinetics of docetaxel and multiple correlative studies were carried out.

**Results:**

Twenty‐four participants were enrolled, 18 in a dose escalation cohort to define the maximum tolerated dose (MTD), and six in a pharmacokinetic cohort. Docetaxel/ADT plus 150 mg/day synthetic lycopene resulted in dose‐limiting toxicity (pulmonary embolus) in one out of 12 participants with an estimated probability of .106 and thus was chosen as the MTD. Lycopene increased the AUC_inf_ and *C*_max_ of plasma docetaxel by 9.5% and 15.1%, respectively. Correlative studies showed dose‐related changes in circulating endothelial cells and vascular endothelial growth factor A, and reduction in insulin‐like growth factor 1R phosphorylation, associated with lycopene therapy.

**Conclusions:**

The combination of docetaxel/ADT and synthetic lycopene has low toxicity and favourable pharmacokinetics. The effects of lycopene on biomarkers provide additional support for the toxicity‐dependent MTD definition.

**Highlights:**

- The maximum tolerated dose was identified as 150 mg/day of lycopene in combination with docetaxel/ADT for the treatment of metastatic prostate cancer patients.
- Small increases in plasma exposure to docetaxel were observed with lycopene co‐administration.
- Mechanistically significant effects were seen on angiogenesis and insulin‐like growth factor 1 signalling by lycopene co‐administration with docetaxel/ADT.

## Graphical abstract

Lycopene is a commonly used dietary supplement among prostate cancer patients. No clinical trial combining docetaxel with lycopene has been reported, possibly due to the fear of antagonism. Our preclinical studies reported that dietary lycopene enhanced the anti‐prostate tumour activity of docetaxel. We performed a phase I study to investigate the maximum tolerated dose (MTD), safety and pharmacokinetics of synthetic lycopene (in combination with docetaxel) and their effects on biomarkers related to insulin‐like growth factor 1 (IGF‐1) signalling and angiogenesis in chemotherapy‐naïve men with metastatic castration‐resistant prostate cancer. The MTD was identified as 150 mg/day lycopene. Small increases in plasma exposure to docetaxel were observed with lycopene co‐administration. Mechanistically significant effects were seen on angiogenesis and IGF‐1 signalling.

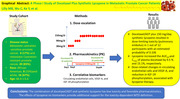

## 1 INTRODUCTION

Since the publication of the TAX327 study in 2004, docetaxel 75 mg/m^2^ every 3 weeks (in combination with prednisone 5 mg twice daily and androgen deprivation therapy [ADT]) has been the standard first‐line chemotherapy regimen for metastatic castration‐resistant prostate cancer (mCRPC), based on improvement in overall survival.^1^ More recently, the CHAARTED study^2^ provided the first demonstration that a similar dose of docetaxel (in combination prednisone and ADT) also improves progression‐free and overall survival for patients with high‐volume, metastatic castration‐sensitive prostate cancer (mCSPC). However, the improvement in overall survival remains modest (increased overall survival of 2.4 months for mCRPC,^1^ and 16.8 months for mCSPC^2^). Furthermore, docetaxel toxicity remains problematic, especially cytopenias, neuropathy and fluid retention.

To improve the therapeutic index for docetaxel chemotherapy, many investigators have performed phase 1−3 clinical trials of docetaxel (usually with prednisone and ADT) in combination with other agents. For patients with mCSPC, the addition of abiraterone^3^ or darolutamide^4^ to docetaxel (plus prednisone and ADT) has improved overall survival compared with survival seen for docetaxel (plus prednisone and ADT) alone. For mCRPC patients, numerous classes of agents have been examined in combination with docetaxel, often with prednisone and ADT. These classes have included targeted agents, immunotherapy, cytotoxic drugs, radionuclides and anti‐angiogenesis agents. None have shown a positive overall survival effect in randomised trials in spite of biochemical effects, and even increases in progression‐free survival, in early‐phase trials. These efforts have been reviewed in several papers.^5^, ^6^

One class of agents that could be combined with docetaxel might be natural products, such as metformin, vitamins, flavonoids, retinoids and carotenoids. Such agents are generally well tolerated and often show anti‐cancer effects with in vitro or in vivo models. However, with the exceptions of calcitriol,^7^ curcumin^8^ and metformin,^9^ few recent randomised clinical trials have been performed. The trials to date have generated much controversy regarding the optimum form, dose and methods for validating biochemical and biological effects while failing to demonstrate improved overall survival.

Lycopene, the red pigment rich in tomatoes, watermelon and other fruits, is the main carotenoid found in the human body.^10^ The initial support for the use of lycopene in prostate cancer prevention and treatment has come from very large retrospective and prospective epidemiological studies, which suggested that higher dietary intakes of tomatoes and tomato products are associated with a reduced risk for developing prostate cancer.^11^, ^12^ Lycopene was found to accumulate more in prostate tissue compared to other organ sites,^10^ and a high plasma level of lycopene was associated with a 25% reduced risk of prostate cancer.^13^ However, the association between lycopene intake and prostate cancer risk was inconsistent in epidemiological studies that were conducted in different settings (i.e., pre‐prostate‐specific antigen [PSA] and post‐PSA eras).^11^, ^12^, ^13^, ^14^, ^15^, ^16^, ^17^ Epidemiological studies in the pre‐PSA era showed an inverse relationship between lycopene intake and prostate cancer incidence, whereas some studies in the PSA era showed no association. This inconsistency has been thought to be, at least in part, due to the heterogeneity of prostate cancer.^17^, ^18^ Subgroup analysis revealed that the association between lycopene intake and prostate cancer was stronger in patients with lethal or higher‐risk prostate cancer than in those with indolent prostate cancer.^17^, ^18^, ^19^

Lycopene has been investigated to treat established prostate cancer in several phase I and II pilot studies in the neoadjuvant setting, and in patients with biochemical relapse, metastatic prostate cancer or castration‐resistant prostate cancer.^20^, ^21^, ^22^, ^23^, ^24^, ^25^ These small studies did not support the use of lycopene as a single agent for the treatment of prostate cancer. Consumption of tomato products and lycopene supplements remains widespread among prostate cancer patients, including those undergoing docetaxel‐based chemotherapy.^26^ It remains unknown whether the consumption of dietary lycopene would affect the safety and efficacy of docetaxel‐based chemotherapy in clinics.

We have shown that lycopene acts synergistically with docetaxel to reduce the viability of human prostate cancer cells in an insulin‐like growth factor 1 (IGF‐1)‐dependent manner and to enhance the anti‐tumour efficacy of docetaxel in a xenograft mouse model of prostate cancer.^27^ Lycopene can also exert its anti‐proliferative effects on prostate cancer cells through its antioxidant properties and suppression of androgen and IGF‐1 signalling pathways.^28^ IGF‐1 signalling is a major survival pathway for cancer cells and is involved in the development of castration‐resistant prostate cancer and docetaxel resistance via interaction with androgen signalling.^29^, ^30^ Based on these results, we hypothesised that lycopene would act synergistically with docetaxel to kill prostate cancer cells in patients by the involvement of the IGF‐1 signalling axis. We have conducted a phase I clinical trial in metastatic prostate cancer patients to identify an optimum dose of lycopene for its combination with docetaxel, to evaluate the safety of the combination and to document the pharmacokinetic (PK) effects of docetaxel and lycopene during combination therapy. We also determined the effects of lycopene plus docetaxel therapy on multiple biomarkers related to IGF‐1 signalling and angiogenesis.

## 2 PATIENTS AND TRIAL METHODS

### 2.1 Protocol design

The study was a phase I clinical trial of docetaxel in combination with synthetic lycopene (BASF Chemicals) in patients who were planning to receive docetaxel as the standard of care for either mCRPC or mCSPC. The trial involved two clinical sites, the Medical University of South Carolina (MUSC) and Ralph H. Johnson Veterans Administration Medical Center in Charleston, South Carolina. Institutional review boards at each site approved the study protocol. The study was conducted according to the Declaration of Helsinki principles and Good Clinical Practice in accordance with International Conference on Harmonisation guidelines. Written informed consent was obtained from all subjects. The trial has been registered (http://www.clinicaltrials.gov) and assigned registration number NCT01949519.

### 2.2 Study population

Participants (aged ≥ 18 years) had mCRPC or mCSPC, ongoing medical or surgical castration with a serum testosterone level of <50 ng/dL, and no prior docetaxel chemotherapy for at least 1 year. Androgen deprivation was maintained throughout the trial. Participants were required to have an ECOG performance status of 0−2, absolute neutrophil count >1500/µL, haemoglobin of >8.0 g/dL and platelets >100 000/µL. In addition, subjects were required to have adequate hepatic and renal function within 14 days of initiation of treatment. Patients were ineligible for enrollment if they had evidence of uncontrolled brain or spinal cord metastases; grade 2 neuropathy, coagulopathy, congestive heart failure or myocardial infarction within the previous 6 months; history of allergy or hypersensitivity to any component of the study drugs; or any uncontrolled concurrent medical condition, which would increase the risk of serious toxicity from the study drugs. Furthermore, patients were ineligible if they concurrently used any vitamin, herb or mineral supplements containing lycopene for at least 14 days prior to the start of therapy.

### 2.3 Treatments

#### 2.3.1 Treatment agents

Synthetic lycopene powder (LycoVit 10/CWD[S]) was donated by BASF Chemicals. The powder was packaged into capsules containing 30 mg of active lycopene by the Investigational Drug Pharmacy at the Hollings Cancer Center, MUSC. Lycopene was administered orally once every morning beginning on day 1 (14 days prior to the first dose of docetaxel) for the duration of study participation. The Food and Drug Administration has determined that this form of synthetic lycopene is biologically equivalent to the natural molecule and has placed it on the generally‐recognised‐as‐safe list.

Commercial docetaxel was obtained by the treating site from their usual sources. Docetaxel was started on day 15 and continued every 21 days thereafter (days 36, 57, 78, etc.) for four cycles of treatment. For mCRPC patients with clinical benefit, docetaxel and lycopene could be continued until disease progression (PCWG 2 criteria [26]), limiting toxicity or withdrawal of consent. Patients with mCSPC could continue study treatment for a maximum of six cycles as validated by the CHAARTED study.^2^ Docetaxel dose adjustment was not permitted. The taxane was given as a 1 h infusion, and supportive care, including glucocorticoids, was at the discretion of the treating physician. Prophylactic pegfilgrastim was permitted for patients with chronically instrumented urinary tracts, recurrent infections, or clinical features associated with an increased risk of neutropenic fever.^31^ Nine of 24 enrolled subjects received at least one dose of pegfilgrastim. In all subjects, ADT had been started at least 1 month prior to trial enrollment and was continued throughout study participation, using the treating physician's preferred Gonadotropin‐releasing hormone agonist/antagonist.

#### 2.3.2 Dose escalation cohort

Three dose levels of synthetic lycopene (30, 90 and 150 mg/day) were planned. The escalation rules, patient allocation and definition of maximum tolerated dose (MTD) are described below under statistical methods. Eventually, 18 patients were enrolled in the dose escalation cohort.

#### 2.3.3 Pharmacokinetic cohort

Six additional subjects were recruited to a PK cohort at the MTD lycopene dose. Docetaxel was started on day 1 and continued every 21 days thereafter. Lycopene administration started on day 2. This allowed the collection of baseline docetaxel PK samples in the absence of lycopene.

### 2.4 Safety assessments

Safety and tolerability were determined by assessment of adverse events, concomitant medications, physical examinations, ECOG performance status and laboratory studies. The severity grade of toxicities was classified using the National Cancer Institute Common Terminology Criteria for Adverse Events, version 4.0. The MTD was defined as one or more of the following, occurring up to day 36: febrile neutropenia that occurred in a cycle during which pegfilgrastim was administered, neutropenia (absolute neutrophil count <500/µL) that persisted for more than 7 days in a cycle during which pegfilgrastim was administered, grade 4 thrombocytopenia, grade 3−4 non‐haematologic toxicity, which did not resolve to grade 2 or less by 28 days after chemotherapy administration.

### 2.5 Statistical methods for phase I trial

#### 2.5.1 Study design

We used the Bayesian‐model‐averaging continual reassessment method (BMA‐CRM)^32^, ^33^ to find the MTD of lycopene combined with a fixed dose of docetaxel. The target toxicity rate of .2, and the MTD were determined from the three doses (30, 90 and 150 mg) per daily administration, hereafter referred to as dose levels {1, 2, 3}. Patients were enrolled in a cohort size of three, and the starting dose was level 1. For the implementation of the BMA‐CRM, two probability skeletons were considered (.05, .1, .2) and (.08, .2, .4), under the model with $\pi_{k,j}=\mathrm{Prob}\left(\mathrm{toxicity}\;\mathrm{at}\;\mathrm{dose}\;\mathrm{level}\;j|\mathrm{skeleton}\;p_{k,j}\right)={p_{k,j}}^{\exp(\alpha )}$, for k=1,2 (skeleton or model) and j=1,2,3 (dose level), where the prior was α∼N(0,2). Under the BMA‐CRM model, $\pi_{j}=\mathrm{probability}\;\mathrm{of}\;\mathrm{toxicity}\;\mathrm{at}\;\mathrm{dose}\;\mathrm{level}\;j,\;j=1,2,3$ was a Bayesian data‐weighted mixture of $\pi_{1,j}$ and $\pi_{2,j}$. The trial would have been terminated early if the lowest dose level was found to be excessively toxic, formally if $\mathrm{Pr}(\pi_{1}>.20|\mathrm{data})>.85$.

#### 2.5.2 Accrual

Toxicities were monitored for each enrolled subject, and it was documented whether or not a dose‐limiting toxicity (DLT) had been observed between days 1 and 36 of the study. The probability of toxicity at each dose level was calculated based on the pre‐specified BMA‐CRM model using data from the previously enrolled subjects, and the dosage for the next enrolled subject was assigned. This process was repeated until 18 subjects had been accrued into the study. The final probability of toxicity at each dose level was calculated using data from the 18 subjects. The MTD was determined by the highest dosage with the final probability of toxicity that was closest to the pre‐specified target toxicity probability of .20. The frequency count of the number of unique patients with an adverse event having the worst grade was calculated and reported.

### 2.6 Correlative studies

#### 2.6.1 Pharmacokinetic evaluations

Plasma samples for docetaxel PK evaluation were collected from six patients in the PK cohort, at the start of cycle 1 (coincident with the first dose of docetaxel, and before initiation of lycopene dosing) and cycle 4 (after 84 consecutive days of lycopene intake). In these subjects, there was no 15‐day period of lycopene‐only treatment. The first dose of docetaxel was administered on day 1 and lycopene was given daily from day 2 until the end of the study. The plasma collection times at each cycle were pre‐infusion and at .5, .75, 1, 2, 4, 8, 12 and 24 h after the start of the docetaxel infusions. Validated high‐performance liquid chromatography methods were used to measure plasma concentrations of docetaxel and lycopene.^34^, ^35^ The lower limit of quantification of docetaxel and lycopene was 8.5 ng/mL and .1 µM, respectively. Docetaxel PK parameters were calculated from concentration versus time data using a non‐compartment model and WinNonlin software (version 4.1; Pharsight Corporation). Docetaxel PK data without (day 1) or with (day 84) lycopene was analysed by comparison of dose‐normalised maximum plasma concentration (*C*_max_), the area under the plasma concentration–time curve (AUC) from time zero to last measurable plasma concentration (AUC_last_), and AUC from time zero extrapolated to infinite time (AUC_inf_) between the two cycles.

#### 2.6.2 Angiogenesis evaluations

Vascular endothelial growth factor A (VEGF‐A) was measured on Ethylenediaminetetraacetic acid plasma from subjects 1−12 of the dose escalation cohort, with a commercial sandwich ELISA kit (R&D Systems). Circulating endothelial cells (CECs) were measured with an 11‐colour flow cytometry scheme using a method adopted from a previously published protocol.^36^ The definition for CECs was viable cells (alive, intact nucleus) that were positive for surface CD31 and CD34, and that were negative for haematopoietic marker CD45. Additional exploratory markers were CD105 and CD146. CEC numbers were reported as cells/4.0 mL of whole blood and were normalised to the amount present in blood from day 1, prior to the first dose of lycopene. Both VEGF‐A and CECs were measured on day 1 (pre‐lycopene treatment), and on days 15, 36, 57, 78 and 99. Samples were collected before the scheduled docetaxel dose for that day.

Both VEGF‐A and CEC levels were analysed using linear mixed‐effects regression models with a subject‐level random effect included to account for within‐subject correlation. Models were adjusted for the individual's baseline levels in both outcomes. All analyses and hypothesis testing were completed on the log scale and back transformed to estimate fold changes. Analysis was completed in the R environment,^37^ with all tests level *α* = .05. The VEGF‐A levels were near‐linear over time, so time was treated as continuous, and slopes between groups were compared using Wald tests. Levels of CECs were non‐linear over time; therefore, time was treated as a fixed factor in the CEC model, with a time‐by‐dose interaction effect. Pairwise tests of differences between the CEC model‐based estimated means at each time point after zero were completed using the *emmeans* package.^38^ As these analyses are exploratory/hypothesis generating, no multiple comparison adjustments have been made. Simple Bonferroni adjustments would lead to *p*‐value cutoffs of .01 (five comparisons) and .0033 (15 comparisons) for the VEGF‐A and CEC analyses, respectively.

#### 2.6.3 Evaluation of insulin‐like growth factor 1 axis

Insulin‐like growth factor binding protein 3 (IGFBP‐3) levels were measured in duplicate by sandwich ELISA assays using kits from eBioscience in subjects 1–12 of the dose escalation cohort. Plasma samples were collected before lycopene administration (day 1) and before docetaxel infusions on days 15, 36, 57, 78 and 99.

An assay to evaluate the effects of lycopene administration on IGF‐1 signalling was developed. The methods and performance of this assay are described in Figure S1. Briefly, ligand‐dependent phosphorylation of the IGF‐1R was estimated using an ex vivo assay that utilised peripheral blood mononuclear cells (PBMCs). Cells from lycopene‐treated patients were stimulated ex vivo with recombinant human (rh)IGF‐1. The cells were then lysed, and the quantity of phosphoIGF‐1R(Y1131) was estimated by an ELISA assay (Cell Signalling Technologies). Ligand‐dependent phosphorylation was defined as the ratio of pIGF‐1R(Y1131) in ligand‐stimulated PBMCs to the same parameter for unstimulated PBMCs. Fold change in ligand‐dependent phosphorylation over time was determined by comparing the results for a treatment day to the level of ligand‐dependent phosphorylation on day 1. Since the number of cases was small, it was difficult to demonstrate that the data were normally distributed. Thus, the changes in the level of ligand‐dependent phosphorylation over time were analysed by both parametric and non‐parametric methods. This assay was performed on PBMCs from 13 subjects in the escalation cohort, before and at 15, 36, 57, 78 and 99 days after initiation of lycopene therapy. One‐tailed parametric and non‐parametric paired tests were utilised to determine if lycopene treatment reduced the ligand‐dependent phosphorylation of IGF‐1R at Y1131 in the PBMCs of subjects taking lycopene in the escalation cohort.

## 3 RESULTS

### 3.1 Study population

Between December 2013 and September 2015, 24 subjects received lycopene and docetaxel on this protocol. Eighteen were enrolled in the dose escalation cohort to determine the MTD. An additional six participants were enrolled for detailed PK studies at the MTD (PK cohort). One participant in the 150 mg lycopene group withdrew consent after the first dose of docetaxel. Therefore, the results from 23 participants are reported here. Characteristics for the 24 enrolled subjects are summarised in Table 1.

**TABLE 1:** Characteristics of study subjects.

	Lycopene				
	Dose‐escalation subjects^a^			PK subjects^b^	
Variable	30 mg PO (*N* = 3)	90 mg PO (*N* = 3)	150 mg PO (*N* = 12)	150 mg PO (*N* = 6)	Total (*N* = 24)
Age, mean (SD)	69.7 (4.7)	67.7 (4)	63.4 (7.4)	70.2 (2.3)	66.4 (6.4)
Median	68	67	63.5	69.5	67.5
Min, max	66, 75	64, 72	49, 74	68, 73	Age, 75
Race					
African American	1 (33%)	2 (67%)	3 (25%)	2 (33%)	8 (33%)
White	2 (67%)	1 (33%)	9 (75%)	4 (67%)	16 (67%)
Non‐Hispanic	3 (100%)	3 (100%)	12 (100%)	6 (100%)	24 (100%)
Disease status					
mCRPC	3 (100%)	3 (100%)	10 (84%)	4 (67%)	20 (83%)
mCSPC	0	0	2 (16%)	2 (33%)	4 (17%)
Withdrew consent	0	0	1	0	1 (4%)
Evaluable	3	3	11	6	23 (96%)

Abbreviations: mCRPC, metastatic castration‐resistant prostate cancer; mCSPC, metastatic castration‐sensitive prostate cancer; MTD, maximum tolerated dose; PK, pharmacokinetic; SD, standard deviation.

^a^ Eighteen subjects were enrolled for determination of the MTD (non‐PK subjects).

^b^ Six subjects were enrolled for detailed PK studies at the MTD (PK subjects).

### 3.2 Drug treatment and compliance

Four patients with castration‐sensitive disease received only six cycles of the combination regimen per the CHAARTED study.^2^ Twenty patients with castration‐resistant disease were treated with the combination regimen until the development of toxicity, withdrawal of consent or disease worsening. The median duration of lycopene and docetaxel combination treatment was 105 days (range, 62–287 days). The median number of docetaxel cycles was 6.0 (range, 3.0–13.0). The median duration of lycopene intake was 120 days (range, 62–302). Patients treated in the PK cohort had a shorter duration of lycopene and docetaxel treatment (median of 101 vs. 147 days) because of the higher proportion of subjects with mCSPC. One patient in the 150 mg/day dose level of the dose identification cohort withdrew consent after the first dose of docetaxel. He received no further lycopene and none of his data were used for analysis.

### 3.3 Safety and dose‐limiting toxicities

Patients in the dose escalation cohort took lycopene alone as a lead‐in treatment for 2 weeks starting on day 1. No participant reported any adverse event during that time. Adverse events were experienced during combination therapy by all participants. These adverse events most commonly were anaemia (86.9%), fatigue (82.6%) and constipation (65.2%) at any grade (Table 2). Eleven of 23 patients (47.8%) reported at least one grade 3 or higher adverse event (Table 2). Neutropenia (65.2%) and infections (21.9%) were the most common grade 3 or higher toxicities. Eight serious adverse events (SAEs), involving hospitalisation or potentially life‐threatening complications, were experienced by five patients (21.7%) including two from the 30 mg group and three from the 150 mg group. The SAEs included urinary tract infection, decreased neutrophil count, sepsis, hyperglycaemia and pulmonary embolus.

**TABLE 2:** All toxicities occurring in at least 4% of subjects, compared with TAX327 and FIRSTANA trials.

	TAX327		FIRSTANA		Docetaxel + lycopene	
	% any	% grade 3+	% any	% grade 3+	% any	% grade 3+
Anaemia	66.5	4.9	99.5	5.5	86.9	4.4
Neutropenia	40.9	32	89	78.9	70.0	65.2
Thrombocytopenia	3.4	.6	32.6	1.6	21.9	0
Febrile neutropenia	2.7	2.7	8.3	8.3	8.7	8.7
Peripheral oedema	18.1	.3	20.4	1.6	30.5	0
Sensory/motor neuropathy	30.4	1.8	25.1	2.1	30.5	4.4
Rash/desquamation	6	N/A	5.9	N/A	17.4	N/A
Alopecia	65.1	N/A	39	N/A	17.4	N/A
Nail changes	29.5	N/A	9	N/A	13.0	N/A
Nausea	41	2.7	22.7	.8	47.8	0
Diarrhoea	31.6	2.1	37	2.3	56.6	0
Stomatitis/pharyngitis	19.6	.9	13.7	.8	13.0	0
Vomiting	16.9	1.5	11.6	.8	17.4	0
Cough	12.3	0	9.8	0	13.0	0
Dyspnoea	15.1	2.7	9.6	.3	8.7	0
Fatigue	53.3	4.5	28.9	.3	82.6	4.4
Tearing	9.9	.6	9.6	0	17.4	0
Arthralgia	8.1	.6	8	1	4.4	4.4
Pain in extremity	NA	NA	9.8	1	17.4	0
Constipation	NA	NA	18.1	1	65.2	0
Dysgeusia	18.4	0	18.1	0	21.7	0
Anorexia	16.6	1.2	17.1	.3	21.7	0
Infection	32.2	5.7	7.2	4.1	34.7	21.9
Pulmonary embolus	NA	NA	NA	NA	8.7	8.7
Somnolence	NA	NA	NA	NA	4.4	4.4
Syncope	NA	NA	NA	NA	4.4	4.4
Hyperglycaemia	NA	NA	NA	NA	4.4	4.4

Abbreviation: NA, not available.

The magnitudes of the common AEs were similar to those seen with other docetaxel trials for metastatic prostate cancer (Table 2; 1, 40). Only two toxicities (constipation, grade 3−4 infection) were more than threefold higher than the average or highest values in the comparison trials. All grade 3 and 4 toxicities occurred during combined therapy, and except for pulmonary emboli, were typical of those seen in historical patients receiving docetaxel therapy alone.

**FIGURE 1:**
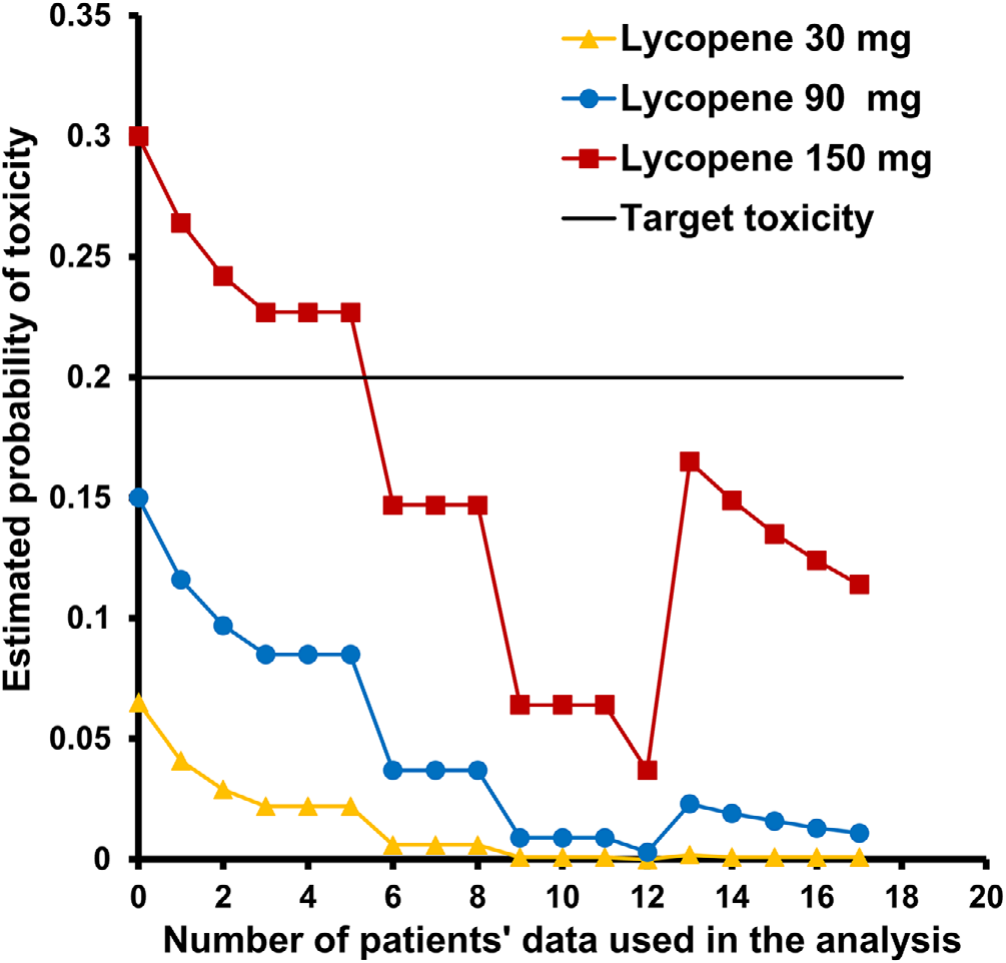
Scatter plot of the estimated probability of toxicity at each dose level. One dose‐limiting toxicity (DLT) (pulmonary embolus) occurred in cohort 3 (150 mg/day). The model predicts that the incidence of DLTs is unlikely to be more than 20% in this cohort; this dose was declared to be the maximum tolerated dose (MTD).

No DLT occurred in the 30 or 90 mg cohorts and the probabilities of toxicity for each dose level were 0, .009 and .106, respectively. One DLT (pulmonary embolus) occurred at dose level 3 (150 mg/day lycopene), and the probability of toxicity was closest to the pre‐specified target value of .20. Therefore, the MTD of synthetic lycopene (in combination with docetaxel and ADT) was determined to be 150 mg/day by the Bayesian continuous reassessment model. The estimated probability of toxicity at each dose level is listed in Table 3, and a scatter plot for the probability of toxicity is shown in Figure 1. Eventually, two subjects were found to have pulmonary emboli in the dose level 3 (150 mg/day lycopene) cohort, one of which was a DLT. The DLT in patient #15 resulted in a hike in the probability of toxicity to .165. After this, there is no more DLT identified in the rest of the patients (#16, #17 and #18), leading to a continued decrease in the probability of toxicity (Figure 1, the red line, and Table 3).

**TABLE 3:** Estimated probability of toxicity at each dose level for individual participants.

Patient ID	No. of patient's data used in the analysis	Probability of toxicity			Probability that the lowest dose is more toxic than the target
		Dose 1	Dose 2	Dose 3	
1	0	.065	.15	.3	.116
2	1	.041	.116	.264	.07
3	2	.029	.097	.242	.046
4	3	.022	.085	.227	.031
5	4	.022	.085	.227	.031
6	5	.022	.085	.227	.031
7	6	.006	.037	.147	.003
8	7	.006	.037	.147	.003
9	8	.006	.037	.147	.003
10	9	.001	.009	.064	0
11	10	.001	.009	.064	0
12	11	.001	.009	.064	0
14	12	0	.003	.037	
15	13	.002	.023	.165	
16	14	.001	.019	.149	
17	15	.001	.016	.135	
18	16	.001	.013	.124	
	17	.001	.011	.114	

### 3.4 Plasma lycopene levels

**FIGURE 2:**
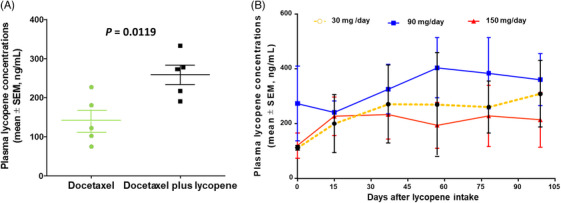
Individual and mean of lycopene concentrations. (A) Mean ± standard error of the mean (SEM) of plasma lycopene concentrations in docetaxel alone versus docetaxel plus lycopene administration (*n* = 5). (B) Mean ± SEM of plasma lycopene concentrations after different durations and doses of lycopene. *N* = 3, 3 and 11 for 30, 90 and 150 mg/day lycopene, respectively.

The plasma concentrations of lycopene were determined for each participant at the baseline (day 0) and after continuously receiving lycopene for 15, 36, 57, 78 and 99 days. Lycopene levels generally increased during treatment. The mean lycopene concentrations in plasma at baseline and after 99 days of treatment were .48 ± .37 and .82 ± .49 µmol/L, respectively (*p* = .0119), an increase of 93.4% (95% confidence interval 25.7%–97.4%) during treatment (Figure 2A). A higher dose of lycopene intake was associated with a shorter time to reach a peak concentration of plasma lycopene (Figure 2B). However, 150 mg/day lycopene did not further increase the peak concentration of lycopene in plasma compared to 30 and 90 mg/day lycopene doses (Figure 2B).

### 3.5 Pharmacokinetics of docetaxel during synthetic lycopene treatment

**FIGURE 3:**
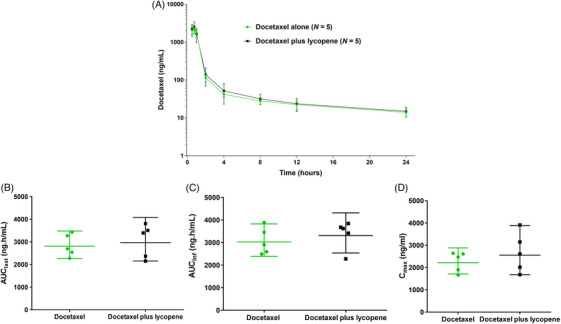
Docetaxel pharmacokinetics before and during lycopene intake. (A) Mean ± standard error of the mean (SEM) plasma docetaxel concentrations over time in docetaxel alone versus docetaxel plus lycopene administration. (B) AUC_last_, (C) AUC_inf_ and (D) *C*_max_. Box plots indicate median and 25%/75% quartiles with whiskers to the last point within 1.5 times the interquartile range.

The plasma concentration–time curve from time zero to the last measurable plasma concentration of docetaxel is shown in Figure 3A. Figure 3B–D shows the comparison of AUC_last_, AUC_inf_ and *C*_max_ values between docetaxel alone and docetaxel plus lycopene. There was no difference in docetaxel PKs with and without lycopene intake (analysis of variance [ANOVA], *p* > .05) (Table 4).

**TABLE 4:** Plasma docetaxel versus docetaxel plus lycopene pharmacokinetic (PK) parameters.

PK parameter	*N*	Geometric mean		Ratio	95% CI
		Docetaxel	Docetaxel plus lycopene		
AUC_last_ (ng h/mL)	5	2810	2968	1.056	(.797–1.399)
AUC_inf_ (ng h/mL)	5	3024	3311	1.095	(.789–1.519)
*C*_max_ (ng/mL)	5	2810	2968	1.151	(.888–1.491)

Abbreviations: AUC, area under the curve; CI, confidence interval.

### 3.6 Angiogenesis markers

#### 3.6.1 Plasma VEGF‐A level

**FIGURE 4:**
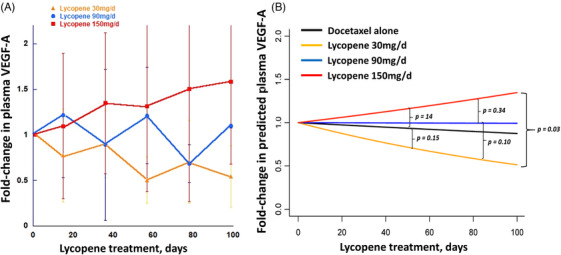
Effects of lycopene and docetaxel on vascular endothelial growth factor A (VEGF‐A) levels. (A) Serial measurements of fold change in VEGF‐A, with pretreatment value = 1. Each point is the mean (±standard deviation [SD]) of measurements from three (30 or 90 mg/day lycopene) or six (150 mg/day lycopene) consecutive subjects. (B) linear regression analysis of data from (A), plus historical control subjects receiving docetaxel alone (six subjects). *p*‐Values show the likelihood of no difference between the indicated slopes.

The mean VEGF‐A level for all subjects at the start of treatment was 352.9 ± 87.0 pg/mL (mean ± SEM). The VEGF‐A level showed a biphasic response to the lycopene dose. Over time, VEGF‐A levels tended to decrease in subjects receiving 30 mg/day of lycopene and to increase in subjects receiving 150 mg/day. Subjects in the 90 mg/day cohort showed little change in VEGF‐A levels, compared to baseline levels (Figure 4A). To clarify differences in VEGF‐A levels over time, we performed a linear regression analysis, with the addition of data from archived plasma samples from patients who were treated with docetaxel/ADT alone for metastatic prostate cancer (controls, nine samples from six unique patients). Regression lines were fit to each population to model the predicted VEGF‐A levels. Subjects receiving lycopene 30 mg/day were predicted to have lower VEGF‐A levels than historical control subjects receiving docetaxel alone, while subjects treated with 150 mg/day lycopene had higher levels (Figure 4B). Subjects receiving the intermediate 90 mg/day dose were predicted to have VEGF‐A levels similar to those of the historical controls. The difference in predicted VEGF‐A between the 30 and 150 mg/day cohorts was statistically significant (*p* = .03).

#### 3.6.2 Circulating endothelial cells

**FIGURE 5:**
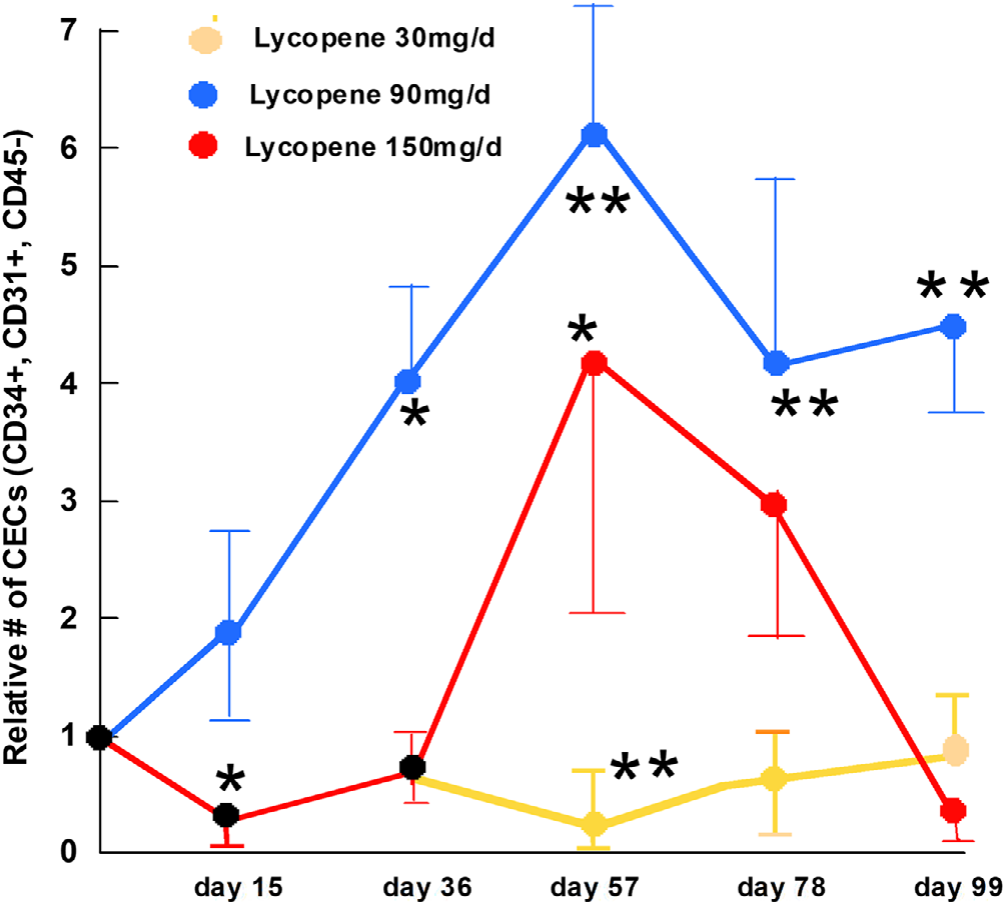
The effects of lycopene and docetaxel co‐administration on circulating endothelial cells (CECs). Fold change in CEC level during lycopene administration, with pretreatment value = 1. Asterisks indicate a significant difference from the day 0 value by paired two‐tailed *t*‐tests.

Before treatment subjects were found to have 788 ± 746 CECs/4 mL of blood (mean ± standard deviation [SD]). CECs also showed a biphasic response to lycopene plus docetaxel treatment (Figure 5). In subjects receiving lycopene at 30 mg/day, the mean number of CECs decreased (compared to pre‐treatment levels) at all time points. At day 15, the reduction was of borderline significance (*p* = .065), while at day 57, the level was significantly reduced compared with day 0 (*p* = .031). Subjects receiving lycopene 90 mg lycopene daily had increased CEC levels at all time points. The increase was statistically significant (*p* < .05 for no difference) at all points. Patients treated with lycopene 150 mg daily had a significant increase in CECs only on day 57, with non‐significant changes on all other days. The CEC nadir for the 30 mg/day lycopene group also occurred on the same day as the CEC apogee for the 90 and 150 mg/day lycopene patients, suggesting mechanistic linkage.

### 3.7 IGF‐1 signalling axis

#### 3.7.1 IGFBP‐3 levels

Serial measurement of plasma IGFBP‐3 levels was performed on days 1, 15, 36, 57, 78 and 99 on 12 consecutive subjects from the dose escalation cohort. The average pretreatment level was 167.1 ± 54.2 ng/mL (mean ± SD). There were no statistically significant differences from the pretreatment level among subjects receiving different doses of lycopene, at any time point.

#### 3.7.2 Ligand‐dependent phosphorylation of IGF‐1R(Y1131)

**FIGURE 6:**
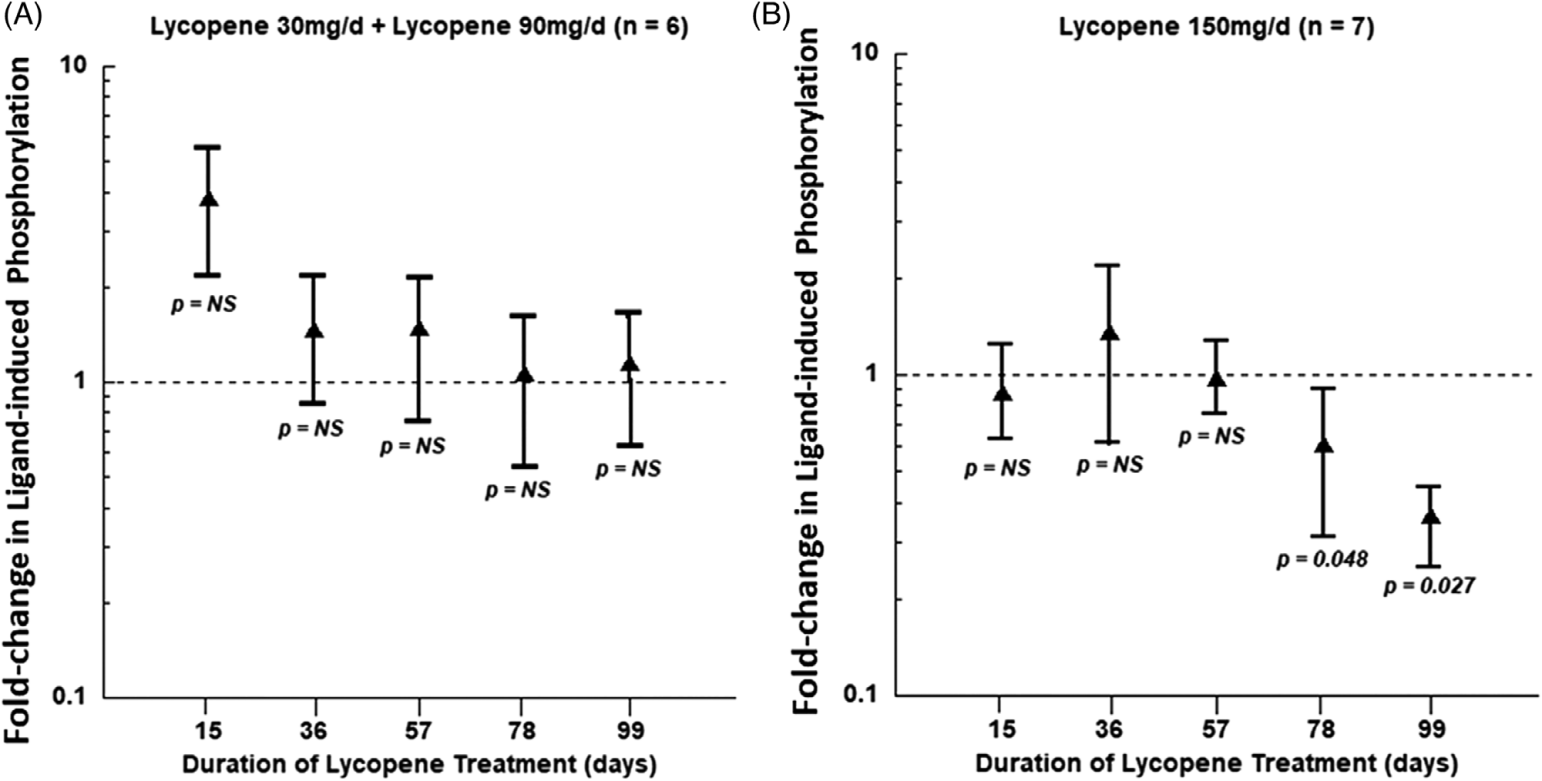
Effects of lycopene and docetaxel co‐administration on ligand‐dependent IGF‐1R(Y1131) phosphorylation. Fold change in ligand‐dependent insulin‐like growth factor 1R (IGF‐1R) phosphorylation in peripheral blood mononuclear cells (PBMCs) with pretreatment value = 1. (A) Pooled values from subjects receiving 30 mg/day (*n* = 3) and 90 mg/day (*n* = 3) lycopene. (B) Pooled values from subjects receiving 150 mg/day lycopene (*n* = 7). Comparison labelled *p* = NS is not significantly different from the pretreatment values, by paired *t*‐tests or Wilcoxon signed‐rank test.

Our pilot data showed that PBMCs from a normal volunteer had reduced ligand‐dependent phosphorylation of the IGF‐1R during synthetic lycopene administration (Figure S1), suggesting that our observation of impaired IGF‐1 signalling in lycopene‐treated cells^27^ could occur in patients as well. Ex vivo stimulation of PBMCs from study subjects resulted in an increase in IGF1‐R(Y1131) phosphorylation by 10.9‐fold (range, 2.6–59.1) above that of unstimulated cells. During study treatment, the amount of ligand‐dependent phosphorylation (ratio of IGF1‐R[Y1131] content with stimulation, compared with unstimulated cells) did not show a consistent change (increase or decrease) in subjects receiving 30 or 90 mg/day of lycopene, either at day 15 (lycopene alone) or at any subsequent day (lycopene plus docetaxel; Figure 6A). In subjects receiving 150 mg of lycopene daily, ligand‐dependent phosphorylation of IGF1‐R(Y1131) did show a borderline decrease at day 78 (*p* = .048 by paired *t*‐test), and a significant decrease at day 99 (*p* = .027 by paired *t*‐test; Figure 6B). Because of the small number of patients, it was not possible to determine if the data were normally distributed for each dose cohort. We therefore pooled the data for the 30 and 90 mg/day lycopene doses (*n* = 6) and examined fold change in ligand‐dependent phosphorylation by the Wilcoxon signed‐rank test. This non‐parametric assay showed no significant difference in ligand‐induced phosphorylation between the start of treatment and any follow‐up day, for subjects receiving 30 or 90 mg/day of lycopene. For the subjects receiving 150 mg/day of lycopene, the Wilcoxon signed‐rank test showed no significant difference between baseline and any follow‐up day, for days 15, 36, 57 and 78. However, for day 99, the non‐parametric test also showed a significant (*p* < .05) reduction in ligand phosphorylation, as was detected by the paired *t*‐test. These data demonstrated that lycopene treatment produces a dose‐dependent reduction in signalling by IGF‐1 through its receptor.

### 3.8 PSA assessments

The purpose of this phase I trial was not to determine the anti‐tumour efficacy of the combination of synthetic lycopene and docetaxel. PSA levels were, however, recorded. The median baseline PSA for the patients (*n* = 23) was 19.2 µg/L (range, .6−405.6). Patients had a median percentage change from baseline to nadir of −72.81% (range, −100.0 to −11.7). A reduction of ≥50% from baseline in PSA was observed in 13 of 23 (56.5%) patients.

## 4 DISCUSSION

This is the first phase I study to evaluate the safety, MTD and PK of lycopene and docetaxel combination regimen for advanced prostate cancer. Synthetic lycopene had little clinical toxicity at doses up to 150 mg/day, in combination with docetaxel. A higher dose of lycopene may not be useful due to a plateau in the plasma level, observed at the dose level of 90 mg/day or higher.

Multiple prior studies show that *trans*‐lycopene is the predominant species in natural sources, whereas *cis*‐lycopene comprises the vast majority of lycopene in the body.^10^ The synthetic lycopene preparation used in our study consists of 77% *trans*‐lycopene and 23% *cis*‐lycopene, approximately the composition of natural tomato paste. Thirty milligrams daily of synthetic lycopene, administered with docetaxel, increased plasma lycopene concentration by approximately 1.6‐fold from baseline. Published studies showed that plasma lycopene levels increased 1.97‐fold in men with prostate cancer who received tomato sauce containing 30 mg/day lycopene.^15^ We did not examine the racemic composition of lycopene in the plasma samples and cannot speculate on whether the plateau in plasma levels reflects absorption effects or a conversion phenomenon. We do note, however, that there are consistent dose‐related effects on several parameters of angiogenesis and cytokine signalling, despite similar plasma lycopene concentration over the tested dose levels (see below). Thus, the plasma lycopene concentrations may not reflect important biologic processes associated with lycopene treatment, and probably should not be the sole metric for deciding on an optimum dose for clinical trials.

Although lycopene has been shown to upregulate the expression of CYP3A4, a major catabolic enzyme for docetaxel,^39^, ^40^ our PK analyses did not show any detrimental drug–drug interaction for the lycopene and docetaxel combination regimen. Instead, we observed that geometric mean docetaxel exposure (AUC_inf_) increased by 9.5% and peak concentration (*C*_max_) increased by 15.1% with co‐administration of lycopene and docetaxel. This may be partly because docetaxel is insensitive to CYP3A4 enzyme induction effects through intravenous administration and is a drug with a high extraction ratio.

The major toxicities were similar to those seen in the comparison trials of single‐agent docetaxel treatment. One toxicity that was markedly different from the reference trials (threefold or greater difference from the mean or highest value in the comparison trials) was constipation, which was 3.5‐fold higher than the value reported in the FIRSTANA study.^41^ Clearly, the colon epithelium was continually exposed to lycopene since almost all subjects reported ongoing red‐ or orange‐coloured stool during treatment. Lycopene has been reported to prevent toxicities from several cytotoxic drugs, including methotrexate and cyclophosphamide.^42^, ^43^ Docetaxel is well known for producing diarrhea. Possibly lycopene antagonises this agent's mucosal toxicity as well. Constipation has not been previously recognised as a common side effect of lycopene.

We serially examined levels of VEGF‐A and CECs during lycopene therapy based on biochemical, animal and clinical studies showing that lycopene inhibits angiogenesis.^17^, ^44^ Both analyses showed similar, striking dose‐dependent effects of the treatment, suggesting that the processes may be mechanistically related. The plasma VEGF‐A level steadily decreased in subjects receiving 30 mg/day lycopene (plus docetaxel) but increased in patients treated with 150 mg/day, when compared with the pretreatment level. This VEGF‐A response continued to develop during the observation period (99 days). Similarly, the CEC level decreased in subjects receiving 30 mg/day of lycopene and increased in those treated with 90 or 150 mg/day of lycopene. The level of VEGF‐A, and those of CECs decreased during treatment with lycopene 30 mg/day, and increased with higher doses (90 and 150 mg). This pattern of opposite effects at high or low levels of a stressor is typical of hormesis, a fundamental stress response in nearly all forms of life. While hormesis is commonly demonstrable in cell cultures,^45^ only rarely do clinical or animal data document its occurrence in cancer treatment.^46^ Our data demonstrate hormesis in actual patients being treated on a trial. However, differences in dose effects on CECs (higher and longer increases at 90 than 150 mg/day) may reflect a net effect of multiple biologic processes. We note that the plasma level of lycopene was also higher at 90 mg/day than at 150 mg/day. Such a difference could modify the development of hormesis. Because of the possibility of antagonising anti‐cancer effects, the choice of dose level is critically important in studies of cancer treatments known to cause hormesis.^47^

Since the VEGF‐A and CEC fluxes depended on the dose of lycopene (docetaxel and ADT were constant), it is important to understand the mechanisms that connect the treatment to these vascular responses. Acute elevation of CECs has been described as an indicator of vascular damage. Examples of efficacious insults include cancer treatment with vascular disrupting agents,^48^ acute deep venous thrombosis,^49^, ^50^ treatment with ineffective chemotherapy,^51^, ^52^ acute coronary syndrome^53^ and microangiopathy.^54^ Elevation of CEC levels may be accompanied by simultaneous increases in VEGF levels.^48^ Our data showed a transient increase in CECs that was greatest at day 57 and returned towards pretreatment level by day 99. In contrast, VEGF‐A levels continued to increase at least until day 99. This sequence suggests that the increase in CECs is an acute response to a lycopene‐induced vascular insult, with the VEGF‐A increase mediating a prolonged healing process. The vascular insult could be a direct toxic effect of lycopene on endothelium, as has been reported from the treatment of cultured cells.^44^ Such endothelial damage could lead to the development of pulmonary emboli, found to be the DLT at 150 mg/day of lycopene.

We have previously shown^27^ that the growth‐inhibitory effect of lycopene on prostate cancer cell lines is dependent on their IGF‐1R levels, and that lycopene directly inhibits IGF‐1‐induced IGF‐1R activation.^27^ Further molecular modeling analysis revealed that lycopene binds to the IGF‐1R kinase domain (IGF‐1RK) and docks within the PQIP (a known IGF‐1RK inhibitor) binding site (data not shown). We have used a model based on PBMCs to translate these mechanistic studies into the clinical setting. Using ligand‐induced phosphorylation of the IGF‐1R as the primary analyte, we found that lycopene reduced IGF‐1 signalling in PBMCs obtained from subjects taking 150 mg/day of lycopene for 78 or 99 days. The kinetics of this process, which did not occur in subjects taking 30 or 90 mg/day, suggest that it is mechanistically distinct from the vascular effects discussed above. These data are supportive of our toxicity data showing the MTD for synthetic lycopene in combination with docetaxel to be 150 mg/day. This dose may produce less vascular damage (CEC elevation) than the 90 mg/day dose and may therefore be suitable for future trials.

We have noted a previous, randomised phase II trial of IGF‐1 blockade for the treatment of advanced prostate cancer.^55^ This study utilised ADT with or without an anti‐IGF‐1R antibody. The primary clinical response objective was not reached. Correlative studies utilising previously validated biomarkers were also negative. These findings raise questions about the biological effects and dose of the intervention. Our studies here report clinically relevant biological effects from lycopene on the IGF‐1R axis at doses that are achievable and tolerable, including in the presence of docetaxel. Lycopene may also have anti‐tumour effects through other mechanisms than IGF‐1R blockade. Antioxidant effects, androgen blockade and angiogenesis inhibition have been noted previously and may be relevant to prostate cancer treatment.

Further trials of lycopene/docetaxel combinations, coupled with appropriate correlative studies, may be warranted in patients with advanced prostate cancer. Such trials could include further study of dose, as well as the addition of anti‐platelet agents or anticoagulants. We note multiple recent studies that identify ‘incidental’ pulmonary emboli in multiple cancer patients, including those with prostate cancer.^56^ These pulmonary emboli were usually identified by prospective use of pulmonary angiography in asymptomatic patients. An expanded phase II trial of lycopene and docetaxel, coupled with pulmonary angiography may help clarify the true incidence of emboli in the target population, and inform the evaluation of risks and benefits of the addition of agents that affect platelets or endothelial function.

## AUTHOR CONTRIBUTIONS

*Conceptualisation*: Michael B. Lilly and Xiaolin Zi. *Methodology*: Chunli Wu, Yu Ke, Wen‐Pin Chen, Adam C. Soloff, Kent Armeson, Noriko N. Yokoyama, Xiaotian Li, Liankun Song, Ying Yuan and Christine E. McLaren. *Resources*: Michael B. Lilly and Xiaolin Zi. *Data curation*: Chunli Wu, Yu Ke, Wen‐Pin Chen, Adam C. Soloff, Kent Armeson, Noriko N. Yokoyama, Xiaotian Li, Liankun Song and Ying Yuan. *Writing—original draft*: Michael B. Lilly and Xiaolin Zi. *Writing—review and editing*: Michael B. Lilly, Christine E. McLaren and Xiaolin Zi. *Supervision*: Michael B. Lilly and Xiaolin Zi. *Project administration*: Michael B. Lilly and Xiaolin Zi. *Funding acquisition*: Michael B. Lilly and Xiaolin Zi. All the authors have read and agreed to the published version of the manuscript.

## CONFLICT OF INTEREST STATEMENT

The authors declare they have no conflicts of interest.

## ETHICS STATEMENT

The trial involved two clinical sites, the MUSC and Ralph H. Johnson Veterans Administration Medical Center in Charleston, South Carolina. Institutional review boards at each site approved the study protocol. Written informed consent was obtained from all subjects.

## STATEMENT OF TRANSLATIONAL RELEVANCE

Lycopene is a commonly used dietary supplement among prostate cancer patients. No clinical trial combining docetaxel with lycopene has been reported, possibly due to the fear of antagonism. Our preclinical studies reported that dietary lycopene enhanced the anti‐prostate tumour activity of docetaxel. We performed a phase I study to investigate the maximum tolerated dose (MTD), safety and pharmacokinetics of synthetic lycopene (in combination with docetaxel) and their effects on biomarkers related to IGF‐1 signalling and angiogenesis in chemotherapy‐naïve men with mCRPC. The MTD was identified as 150 mg/day lycopene. Small increases in plasma exposure to docetaxel were observed with lycopene co‐administration. Mechanistically significant effects were seen on angiogenesis and IGF‐1 signalling.

## Supporting information

Supporting information

## Data Availability

The data generated in this study are available within the article and its supporting data files.
