# Supplementary material for: A phase I study of docetaxel plus synthetic lycopene in metastatic prostate cancer patients
Source: Clin Transl Med. 2024 Mar 21;14(3):e1627. doi: 10.1002/ctm2.1627 (PMC10958125; doi:10.1002/ctm2.1627)
Supplement: Supplementary file 1 — Supporting information [file CTM2-14-e1627-s001.docx]

**Supplementary Figure S1. Measurement of IGF-1R (Y1131) phosphorylation in PBMNCs after *ex vivo* ligand treatment.** Our previously published data (27) indicated that lycopene binds to the IGF-1R and blocks ligand-dependent signaling. A previous (negative) trial of ADT plus an anti-IGF-1R monoclonal antibody (cixutumumab) in patients with advanced prostate cancer was negative for the postulated clinical effect (31). However, the correlative biomarker studies were also negative, raising questions about the appropriateness of the antibody dose and schedule. We therefore developed an assay to assess ligand-dependent phosphorylation of the IGF-1R in a clinical setting. The assay used PBMCs isolated from patients or volunteers taking lycopene at doses used in the trial. The primary analyte was phosphorylation of the IGF-1R on Y1131, measured by ELISA on lysates of PBMCs treated (or not) with recombinant human IGF-1.

Assay Procedure.

1. Whole blood (10mL) is collected into an ACD (acid citrate-dextrose) Vacutainer®. Peripheral blood mononuclear cells are collected by density gradient centrifugation using LSM Lymphoprep® and an Accuspin® 50mL tube (Sigma). The mononuclear cells are collected, washed x 2 with serum-free RPMI1640 medium, then resuspended in 1mL of RPMI 1640 medium + 0.1% BSA. Cells are then aliquoted into 1mL microcentrifuge tubes, 0.5mL of cells per tube per condition.
2. Cells are incubated for 1-2hr at 37C, then rhIGF-1 (Peprotech) is added to 50-500ng/mL final concentration. Incubation continues for 15min at 37C.
3. Cells are then pelleted and washed with 1mL of cold RPMI 1640 medium (no protein).
4. The cell pellet is lysed with 120mcL of Cell Signalling® ELISA buffer containing phosphatase inhibitors and protease inhibitors. The lysate can be stored at -80C or assayed immediately.
5. For total protein measurement follow the BCA method (BioRad).
6. For IGF-1R(Y1131) ELISA (Cell Signalling® #7302) proceed per kit instructions. For a standard, starve adherent, confluent DU145 prostate cancer cells in a T25 flask, with serum-free DMEM containing 0.1% BSA for 12hrs. Remove and replace the medium, and continue incubation for 1-2hrs longer. Recombinant human IGF-1 is then added to a final concentration of 50ng/mL. Incubate for 15min at 37C, then aspirate the medium and wash the cells with cold PBS or serum-free medium. Lyse the cells *in situ* with Cell Signalling® ELISA lysis buffer supplemented with protease and phosphatase inhibitors. The PBMC samples are treated similarly except that they are in 1mL microcentrifuge tubes instead of tissue culture flasks. Lysates for standards can also be stored at -80C or assayed immediately.

7. To perform the ELISA, generally follow the instruction sheet with the kit, but incubate standards and unknowns in the plate overnight at 4C for maximal sensitivity. The *standard* will be used as a series of 2-fold dilutions, being diluted with assay buffer from the ELISA kit. Thaw a tube of cell lysate standard and use 120mcL for a series of 2-fold dilutions into 120mcL of assay buffer. Use at least seven 2-fold dilutions. With a series of 7 dilutions, the most concentrated standard (1:1 dilution) is assigned a value of 64 units. The other standards (2-fold dilutions) are then assigned values of 32, 16, 8, 4, 2, and 1 units. The standard curve is used to ensure that the assay is working as expected, and that the unknown assays fall on the linear part of the dose-response curve. The most concentrated standard (64 units) should have an OD of > 1.0. For each *unknown*, mix 50mcL of assay buffer with 50mcL of lysate. Each test sample is assayed in duplicate. Results will be expressed as X units of pIGF-1R protein per amount of protein in the lysate. The protein is measured by the BCA method, as mcg/mL. Ideally, all samples (pre- and post-lycopene) for a given subject should be assayed at the same time, with the same standard curve.

This is an example that shows a marked reduction in IGF1-stimulated phosphorylation of IGF-1R on Y1131 in PBMCs isolated from a lycopene-treated normal volunteer. In these experiments blood was obtained from a volunteer taking lycopene 150mg/d x 7d. Blood was drawn before and at 40hr, 7d, 12d, and 14d after starting dosing. PBMNCs were isolated, stimulated *ex vivo* with rhIGF-1, and the cells were then lysed. The amount of pIGF-1R(Y1131) was measured by ELISA and normalized to the amount of cellular protein in the lysate. Then the amount of stimulated phosphorylated receptor was compared to the amount of phosphorylated receptor from unstimulated cells, with the latter being assigned a relative value of 1 (= ligand-dependent phosphorylation). Duplicate measurements were made for each of 2 blood samples at each time point, leading to a total of 4 values for each bar. Reduction in ligand-stimulated phosphorylation of the IGF-1R was 50% at 7 days, at which point lycopene dosing was stopped. However, IGF-1R inhibition continued and increased, reaching a nadir of 65% reduction 12 days after the start of dosing (and 5 days after discontinuing treatment). This level of reduction in ligand-stimulated receptor phosphorylation was statistically significant (paired T test, asterisk). Receptor phosphorylation returned to baseline levels over the following 2 days (total 7 days off therapy).

Figure S1


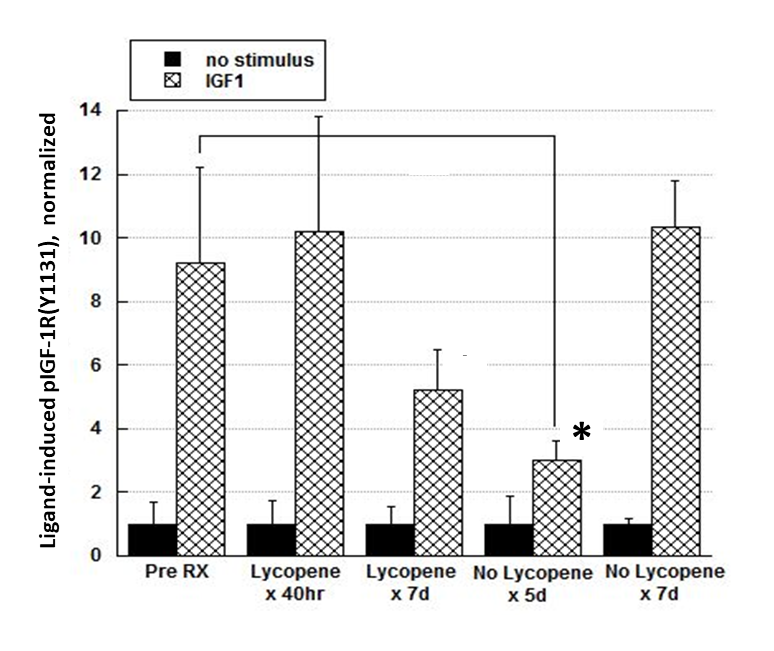


| **Supplementary Table 1.** Representativeness of Study Participants | |
| --- | --- |
| Cancer type(s)/ subtype(s)/stage(s)  /condition | metastatic castration-resistant and castration sensitive prostate cancer (mCRPC and mCSPC) |
| Considerations related to: | |
| Sex | mCRPC and mCSPC, and all other subtypes of prostate cancer, only occur in males. We enrolled only male subjects. |
| Age | Prostate cancer is more likely to develop in older men. The average age of men when they are first diagnosed is about 66. The median age for diagnosis of mCRPC is about 75. The mean age of our subjects was 66.8 (+/- 6.3 SD) years. The range is 49-75. This figure likely is affected by the presence of subjects with mCSPC (newly diagnosed), and the high proportion of African Americans (who develop prostate cancer at earlier ages than do Caucasians). |
| Race/ethnicity | The racial composition of the State of South Carolina is as follows: Caucasian 71%, African American 27%, Hispanic 1.5%, Asian 0.5%. In our study 67% of subjects were Caucasian and 33% were African American. No Asian or Hispanic subjects were enrolled. |
| Overall representativeness of study population | The study population is highly representative of the disease type/stages in our population. |
